# Objective Evaluation of Clinical Actionability for Genes Involved in Myopathies: 63 Genes with a Medical Value for Patient Care

**DOI:** 10.3390/ijms23158506

**Published:** 2022-07-31

**Authors:** Maude Vecten, Emmanuelle Pion, Marc Bartoli, Raul Juntas Morales, Damien Sternberg, John Rendu, Tanya Stojkovic, Cécile Acquaviva Bourdain, Corinne Métay, Isabelle Richard, Mathieu Cerino, Mathieu Milh, Emmanuelle Campana-Salort, Svetlana Gorokhova, Nicolas Levy, Xénia Latypova, Gisèle Bonne, Valérie Biancalana, François Petit, Annamaria Molon, Aurélien Perrin, Pascal Laforêt, Shahram Attarian, Martin Krahn, Mireille Cossée

**Affiliations:** 1 Département de Génétique, Hôpital Bichat-Claude-Bernard, APHP, 75018 Paris, France; maude.vecten@gmail.com; 2 INSERM, Marseille Medical Genetics, U1251, Aix-Marseille Université, 13385 Marseille, France; marc.bartoli@univ-amu.fr (M.B.); mathieu.cerino@ap-hm.fr (M.C.); mathieu.milh@ap-hm.fr (M.M.); emmanuelle.salort-campana@ap-hm.fr (E.C.-S.); svetlana.gorokhova@univ-amu.fr (S.G.); nicolas.levy@univ-amu.fr (N.L.); 3 Laboratoire de Génétique Moléculaire, CHU de Montpellier, 34093 Montpellier, France; emmanuelle.pion@ext.inserm.fr (E.P.); aurelien.perrin@ext.inserm.fr (A.P.); 4 Filnemus, Assistance Publique, Hôpitaux de Marseille, 13354 Marseille, France; annamaria.molon@ap-hm.fr; 5 Département de Neurologie, Hôpital Gui de Chauliac, CHU de Montpellier, 34295 Montpellier, France; rjuntas@vhebron.net; 6 Service de Biochimie Métabolique, Hôpitaux Universitaires Pitié Salpétière, APHP, 75651 Paris, France; damien.sternberg@aphp.fr; 7 Laboratoire de Génétique Moléculaire, CHU de Grenoble et des Alpes, 38043 Grenoble, France; Jrendu@chu-grenoble.fr (J.R.); xenia.latypova@gmail.com (X.L.); 8 APHP, Service de Neuromyologie, Centre de Référence Maladies Neuromusculaires Paris-Est, GH Pitié-Salpêtrière, 75651 Paris, France; stojkovic.tanya@aphp.fr; 9 Centre de Recherche en Myologie, Institut de myologie, Sorbonne Université, Inserm, 75013 Paris, France; g.bonne@institut-myologie.org; 10 Service Biochimie et Biologie Moléculaire Grand Est-UM Maladies Héréditaires du Métabolisme, HCL, 69002 Lyon, France; cecile.acquaviva-bourdain@chu-lyon.fr; 11 UF Cardiogénétique et Myogénétique et Cellulaire, Centre de Génétique moléculaire et chromosomique, Hôpitaux Universitaire Pitié-salpétière, APHP, 75651 Paris, France; corinne.metay@aphp.fr; 12 Centre de Recherche Généthon, Institut des Biothérapies Généthon, 91000 Paris, France; richard@genethon.fr; 13 Laboratoire de Biochimie, Hôpital de la Conception, APHM, 13005 Marseille, France; 14 Service de Neuropédiatrie, Hôpital Timone Enfants, APHM, 13385 Marseille, France; 15 Département de Génétique Médicale, Hôpital Timone Enfants, APHM, 13385 Marseille, France; 16 Laboratoire de Diagnostic Génétique CHU de Strasbourg, 67091 Strasbourg, France; valerie.biancalana@chru-strasbourg.fr; 17 Laboratoire de Génétique Moléculaire Service de Biochimie et Hormonologie Hôpital Antoine Béclère, APHP, 92140 Clamart, France; francois.petit2@aphp.fr; 18 PhyMedExp, Physiologie et Médecine Expérimentale du cœur et des Muscles, Université de Montpellier, Inserm U1046, CNRS UMR 9214, 34295 Montpellier, France; 19 Neurology Department, Nord/Est/Ile de France Neuromuscular Center, Raymond Poincaré Hospital, APHP, Garches, FHU PHENIX, INSERM U1179 INSERM, Université Versailles Saint Quentin en Yvelines, Paris-Saclay, 92380 Paris, France; pascal.laforet@aphp.fr; 20 Service de Neurologie, FILNEMUS, Hôpital La Timone, CHU de Marseille, 13385 Marseille, France; shahram.attarian@ap-hm.fr

**Keywords:** genetics, myopathy, actionability, next generation sequencing, diagnostic

## Abstract

The implementation of high-throughput diagnostic sequencing has led to the generation of large amounts of mutational data, making their interpretation more complex and responsible for long delays. It has been important to prioritize certain analyses, particularly those of “actionable” genes in diagnostic situations, involving specific treatment and/or management. In our project, we carried out an objective assessment of the clinical actionability of genes involved in myopathies, for which only few data obtained methodologically exist to date. Using the ClinGen Actionability criteria, we scored the clinical actionability of all 199 genes implicated in myopathies published by FILNEMUS for the “National French consensus on gene Lists for the diagnosis of myopathies using next generation sequencing”. We objectified that 63 myopathy genes were actionable with the currently available data. Among the 36 myopathy genes with the highest actionability scores, only 8 had been scored to date by ClinGen. The data obtained through these methodological tools are an important resource for strategic choices in diagnostic approaches and the management of genetic myopathies. The clinical actionability of genes has to be considered as an evolving concept, in relation to progresses in disease knowledge and therapeutic approaches.

## 1. Introduction

High-throughput sequencing revolutionized the possibilities of genetic analysis by allowing the simultaneous mutational screening of several genes, progressively extending to all the genes through exome and genome sequencing [1]. These technologies lead to the potential for the recognition of secondary findings unrelated to the indication for ordering the sequencing, but of medical value for patient care [2]. The concept of “actionable genes” has emerged in this context for diagnostic purposes. In 2013, the ACMG drafted initial recommendations on the principle of actionability and published a list of 59 actionable genes [2]. More recently, the ClinGen Actionability Working Group (AWG) proposed a semi-quantitative metric scoring to assess the clinical actionability of genes through four indicators: severity of disease, penetrance/likelihood of disease, effectiveness of intervention, and nature of intervention (https://www.clinicalgenome.org/site/assets/files/2180/actionability_sq_metric.png (accessed on 10 February 2020)) [3], combined with the indication of the level of evidence, to determine best practices regarding secondary findings [4]. Across these topics, the ClinGen AWG scored 213 outcome-intervention pairs from 127 genes associated with 78 disorders [4]. This semi-quantitative measure is set to evolve thanks to the contribution of the entire international community and should be considered as a starting point in the standardization of clinical actionability.

In France, FILNEMUS was the first to publish the “gene-disease” correlations for 199 genes implicated in 223 myopathies [5] using the procedure published by the ClinGen Clinical Validity framework, in order to simplify the molecular diagnosis of myopathies [6]. Despite this, the use of next generation sequencing is faced with results delays, which are problematic for genes with medical value for patient care. ClinGen AWG listed some myopathy genes as actionable, such as the *GAA* gene responsible for Pompe disease, for which the prognosis may improve if the patient is treated quickly especially in its infantile form. However, actionability has not been assessed for all myopathy genes.

Based on the ClinGen AWG recommendations, we carried out a clinical actionability objective assessment of the 199 genes implicated in 223 myopathies reported on the FILNEMUS list.

## 2. Results

We used the semi-quantitative actionability metric established by ClinGen [3,4] to score the clinical actionability of 199 genes implicated in 223 myopathies published by FILNEMUS [5]. We choose to offer an overall score for each gene-associated disorder, to mark the most severe phenotype and not each symptom (cardiac involvement, muscle damage, etc.). The four indicators, severity of disease, penetrance/likelihood of disease, effectiveness of specific intervention, and nature of intervention, were each scored from 0 to 3, from a low level to a high level of actionability [3]. For example, the score severity is 0 if the disease has minimal health impact or no morbidity and 3 if there is sudden death or inevitable death; the score penetrance is 0 if <1% chance or unknown, 3 if >40% chance; effectiveness of specific intervention is 0 if ineffective/no intervention, 3 if highly effective; nature of intervention is 0 if high risk/poorly acceptable/intensive or no intervention, and 3 if low risk/medically acceptable/low intensity intervention [3,4]. All scores have been reviewed by the French FILNEMUS expert-network (clinicians and geneticists).

**Figure 1:**
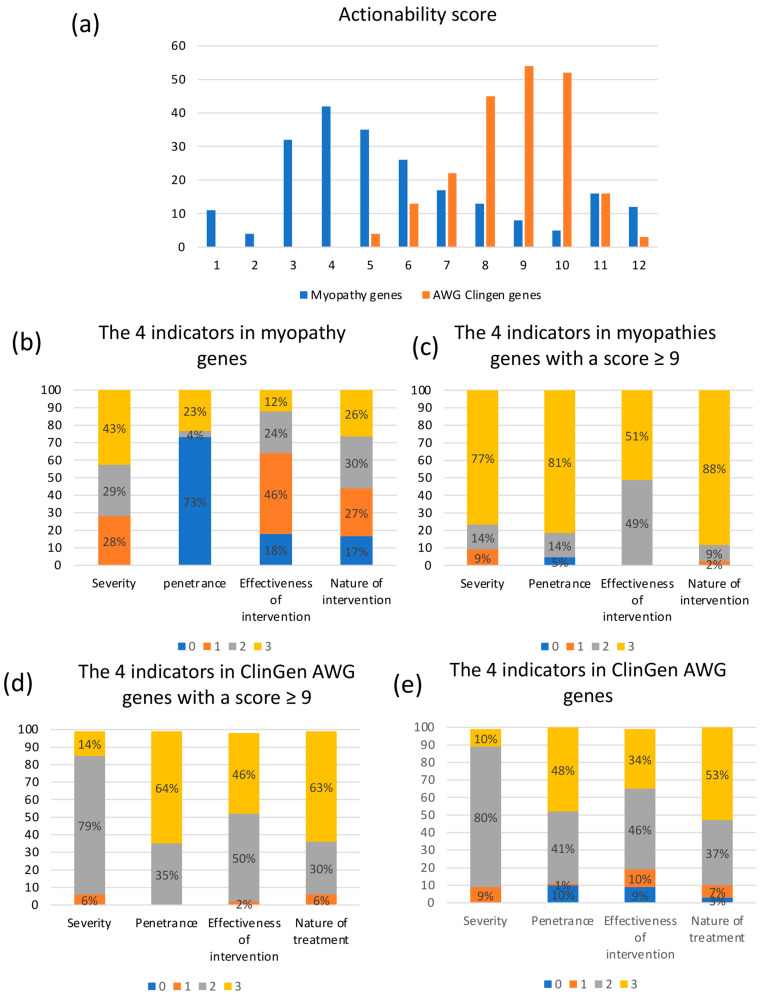
Actionability scores for myopathy genes and ACMG genes.

The 223 myopathy genes/disorders were scored from 1 to 12 (Supplemental Material S1). More than 45% of the scores were distributed between 4 and 6, while the scores for genes/disorders of the ClinGen AWG genes list were between 5 and 12, with >50% of them greater than or equal to 9 (Figure 1a).

The four indicators of actionability were severity of disease, penetrance/likelihood of disease, effectiveness of specific intervention, and nature of intervention. These indicators were each scored from 0 to 3, from a low level to a high level of actionability [3].

a/Total scores for the 223 myopathy genes/disorders (from the 199 genes reported by FILNEMUS [5]) and for the 213 ClinGen AWG genes/outcome-intervention pairs (from the 127 genes scored by ClinGen AWG [4]). b/Distribution of the four indicators for the 223 myopathy genes/disorders. c/Distribution of the four indicators for the 213 ClinGen AWG genes/outcome-intervention pairs. d/Distribution of the four indicators for the 43 myopathy genes/disorders (36 genes) with a global score ≥ 9. e/Distribution of the four indicators for the 125 ClinGen AWG genes/outcome-intervention pairs (111 genes) with a global score ≥ 9.

We evaluated the scores distribution of the four indicators for the myopathy genes/disorders (Figure 1b). Concerning the severity indicator: 43% had very high mortality rates (score 3), 29% had moderate severity (score 2), and 28% low severity (score 1). For the disease penetrance indicator (likelihood): the information was available for only 27% of the myopathy genes/disorders, leading to an artificial score of 0 for 73% of them. For the effectiveness of intervention: 36% were highly or moderate effective (score ≥ 2). The nature of intervention was evenly distributed between the different scores, with a great or high risk (scores 0 and 1) in 44% of cases.

By filtering the two care indicators, effectiveness of specific intervention with a score ≥ 2 and nature of intervention ≥ 1, we retained 63 myopathy genes corresponding to 78 disorders that could be actionable on their management (Table 1). Final scores of actionability of these genes/disorders were between 5 and 12, with the majority ≥ 9 (43/78, 55%). The intervention included either the availability of drugs or a specific cardiac management (defibrillator/pacemaker).

We also evaluated the distribution of actionability scores within the 43 pairs genes/disorders (36 myopathy genes) with higher actionability global scores (≥9) (Figure 1c), of which only 8 had already been scored by ClinGen (Table 1). For the severity indicator: 77% had very high mortality rates, 14% had moderate severity, and 9% low severity. For the disease penetrance indicator: the information was available for 95% of the genes and mostly with full penetrance (81%). The effectiveness of intervention was moderate (score 2) or high (score 3) for all genes. The nature of intervention was moderate or low risk (scores 2 and 3) for 97% of the genes. We then compared these actionability scores with those of ClinGen AWG genes with a global score ≥ 9 (111 genes corresponding to 78 disorders and 125 outcome-intervention pairs) (Figure 1d). We found that the distribution of scores was different between both groups concerning the outcome-related domains (severity and likelihood), with higher scores for myopathy genes (*p* < 0.05). Results for both intervention-related indicators were similar in the two categories of genes.

## 3. Discussion

We applied here, in an exhaustive way for the first time in the field of myopathies, the ClinGen AWG scoring methodological approach.

We encountered a lack of information on the penetrance data of myopathy genes in the literature (for 74% of genes), which was not the case for genes from the ClinGen AWG list genes (Figure 1e) [4]. This lack of information on penetrance led to a “loss” of 1 to 3 final scoring points, that could account for the lower final average actionability score of myopathy genes (score 4–5) compared to the genes scored by the ClinGen AWG (9–10). Noteworthy, an important proportion of myopathies is of autosomal recessive inheritance, with expected complete penetrance. The use of the data collected by the French National Rare Diseases Data Bank (BAMARA) will possibly make it easier in the upcoming years to access data on penetrance for rare myopathies.

Thanks to our scoring work, we have identified 63 myopathy genes (corresponding to 78 disorders) that could be actionable on their management (intervention moderately or highly effective, without a high risk), including 43 genes/disorders with actionability scores ≥ 9. However, only 12 of these myopathy genes were previously scored by ClinGen. Of the 31 genes not scored by ClinGen AWG, 21 are part of congenital myasthenic syndromes (Table 1), for which there are specific treatments, reflecting the value of this scoring work [7].

Some of the 63 myopathy genes/disorders have a lower score of actionability (<9) but are considered to have medical value for patient care. The *CLCN1* gene is a good example in the recessive or dominant myotonia congenita (Table 1). The low global score (7) is due to the modest morbidity (severity scored as 1), but the treatment is highly effective (scored as 3) and acceptable (scored as 3). For some genes associated with several diseases, the increase in the global score for actionability is due to an increase in the severity score. For example, the *SCN4A* gene is associated with an actionability score of 9 in the hyperkalemic periodic paralysis (severity score at 1), compared to a score of 11 in the congenital myasthenic syndrome type 16 (severity score at 3) [8].

Our results will allow the integration of the notion of actionable genes into strategic choices for molecular diagnostic strategies and management of patients with genetic myopathies. We recommend that these 63 genes be analyzed as a priority in the sequencing of gene panels, exomes, or genomes, in order to offer a rapid diagnosis to patients and optimize patient care (treatabolome database in preparation). Furthermore, these genes (at least the 36 with global score ≥ 9) should be considered for a possible addition to the ACMG actionable gene list, in order to assess them as secondary data of medical value for patient care. Of course, patients’ phenotyping remains essential, and the absence of a mutation in this panel of genes does not exclude an intronic mutation in one of these genes. *DMD*, *DM1*, and *DM2* genes were not on the list of FILENEMUS genes analyzed by NGS [5]. However, they should also be considered actionable, given their important consequences on patient management, as well as the *SMN1* gene.

The clinical actionability of genes must be considered as an evolving concept, in relation to progresses in therapeutic approaches, or diagnosis. In recent years, new therapeutic options such as gene therapies have been developed and offer hope for patients with myopathies. It will be important to take these new therapeutic approaches into account in the coming years as several additional myopathic genes will then be considered actionable.

## 4. Materials and Methods

We compared the scores obtained for the myopathy genes/disorders with those of the ClinGen AWG (127 genes corresponding to 78 disorders with 213 outcome-intervention pairs) [4], using the nonparametric Kruskal–Wallis test. Thereafter, we analyzed the distribution of scores of the four indicators for the ClinGen AWG and myopathy genes using the chi-square method that admits differences in group size.

## Figures and Tables

**Table 1:** The 63 myopathy genes with a medical value for patient care. The 63 genes of myopathies were selected based on effectiveness of the treatment with a score ≥ 2 and nature of the intervention ≥ 1. The global score indicated also takes into account the scores of severity and penetrance/likelihood. Genes with a global score ≥ 9 are highlighted in grey. LGMD old/new names according to the LGMD nomenclature are indicated.

Gene	Disorder	Intervention	Effectiveness	Nature of Intervention	Global Score	Clingen Scoring
*ACAD9*	Mitochondrial complex I deficiency due to ACAD9 deficiency	Riboflavine	2	2	6	
*ACADVL*	Acyl-CoA dehydrogenase (very long chain) deficiency (VLCAD deficiency)	Hygienic and dietetic measures +/− triheptanoin acid/Medium cahin triglyceride/N-acetylcystein Prevention of rhabdomyolysis	2	1	9	
*AGL*	Glycogen storage disease type IIIa-GSD IIIa Glycogen storage disease type IIIb-GSD IIIb Glycogen storage disease type IIIc-GSD IIIc Glycogen storage disease type IIId-GSD IIId	Hygienic and dietetic measures with specific diet Vital disease screening because healthy measures can prevent hypoglycaemia and cardiac complication	2	2	6	
*AGRN*	Congenital myasthenic syndrome 8, with pre- and postsynaptic defects	Salbutamol, ephedrine	3	3	12	
*AMPD1*	Myopathy due to myoadenylate deaminase deficiency	Symptomatic treatment/D-ribose	2	1	4	
*BAG3*	Myofibrillar myopathy 6	Symptomatic treatment: Implantable Cardioverter-Defibrillator (ICD)/ventilation	2	3	11	9 AD myopathy myofibrillar (adult)
	Susceptibility to malignant hyperthermia	Avoidance of triggering anesthetics	3	3	10	10 DB malignant hyperthermia susceptibility
	Hypokalemic periodic paralysis, type 1 (mutational hotspots in exons 4, 11, 21, 30)	Acétazolamide, treatment and prevention of paralytic attacks	3	3	9	
*CHAT*	Congenital myasthenic syndrome 6 (presynaptic)	Acetylcholinesterase inhibitors, 3,4-diaminopyridine, salbutamol, ephedrine	3	2	11	
*CHRNA1*	Congenital myasthenic syndrome 1A Congenital myasthenic syndrome 1B Multiple pterygium syndrome, lethal type	Fluoxetine, quinidine	2	3	11	
*CHRNB1*	Congenital myasthenic syndrome 2C, associated with acetylcholine receptor deficiency Congenital myasthenic syndrome 2A + Fetal akinesia deformation sequence	Fluoxetine, quinidine	2	3	11	
*CHRND*	Congenital myasthenic syndrome 3A Congenital myasthenic syndrome 3B Congenital myasthenic syndrome 3C Multiple pterygium syndrome, lethal type	Fluoxetine, quinidine	2	3	11	
*CHRNE*	Congenital myasthenic syndrome 4A, slow-channel	Fluoxetine, quinidine	2	3	11	
	Congenital myasthenic syndrome 4B, fast-channel	Acetylcholinesterase inhibitors, salbutamol, ephedrine, 3,4-diaminopyridine	3	3	12	
	Congenital myasthenic syndrome 4C, associated with acetylcholine receptor deficiency	Acetylcholinesterase inhibitors, 3,4-diaminopyridine, salbutamol, ephedrine	3	3	12	
*CLCN1*	Thomsen Myotonia congenita Becker congenita Myotonia	Mexiletine (side effect including abdominal pain)/lamotrigine/Ranolazine	2	2	7	
	Myotonia congenita (recessive)	Mexiletine, carbamazepine	3	3	7	
	Myotonia congenita (dominant)	Mexiletine, carbamazepine	3	3	7	
*COL13A1*	Congenital myasthenic syndrome 19	3,4-Diaminopyridine Salbutamol	3	3	12	
*COL3A1*	Ehlers-Danlos syndrome, hypermobile	Symptomatic and prophylactic treatment	2	2	10	10 CA Ehlers-Danlos type IV, adult, pediatric
*COLQ*	Congenital myasthenic syndrome 5	Salbutamol and Ephedrine	3	3	12	
*CPT2*	CPT II deficiency, infantile CPT II deficiency, lethal neonatal CPT II deficiency, myopathic, stress-induced	Hygienic and dietetic measures: the need to abandon total diet for a low-fat diet with high carbohydrates +/− L-carnitine. Adult/muscular forms screening utility for rhabdomyolysis prevention (avoid fever, fast and long physical effort). Emergency form is given to the patient	2	2	7	
*DES*	LGMD1E/Myofibrillar myopathy LGMD2R/Myofibrillar myopathy	Cardiac risks prevention/Defibrillator if needed	2	3	8	10 AD myopathy myofibrillar (adult)
	Scapuloperoneal syndrome, neurogenic, Kaeser type	Cardiac risks prevention/Defibrillator if needed	2	3	8	
	Myopathy, myofibrillar, 1	Symptomatic treatment: prevention of cardiac risks. ++ Defibrillator treatment if necessary	2	3	11	
*DOK7*	Congenital myasthenic syndrome 10 Fetal akinesia deformation sequence	Salbutamol, ephedrine	3	3	12	
*DPAGT1*	Congenital myasthenic syndrome 13, with tubular aggregates DPAGT1-CDG/ALG7-CDG	Acetylcholinesterase inhibitors, 3,4-diaminopyridine, salbutamol, ephedrine	3	3	12	
*EMD*	Emery-Dreifuss muscular dystrophy 1, X-linked	Symptomatic treatment +/− orthopedic surgery, pacemaker, heart transplant	2	3	8	7 DC Emery-Dreifuss muscular dystrophy (adult)
*ETFA*	Multiple acyl-CoA dehydrogenase deficiency (MADD; Glutaric aciduria type IIA)	Hygienic diet plus symptomatic treatment	3	3	9	
*ETFB*	Multiple acyl-CoA dehydrogenase deficiency (MADD; Glutaric aciduria type IIB)	Hygienic diet plus symptomatic treatment	3	3	9	
*ETFDH*	Multiple acyl-CoA dehydrogenase deficiency (MADD; Glutaric aciduria type IIC)	Hygienic diet plus +/− riboflavin plus symptomatic treatment	2	1	6	
*FHL1*	Reducing body myopathy, X-linked 1a, severe, infantile or early childhood onset/Emery-Dreifuss muscular dystrophy 6, X-linked/myopathy x-linked with postural atrophy/scapuloperitonael myopathy X-linked	Symptomatic treatment: physiotherapist, orthopaedic surgery (tendon retraction), cardiological treatment + defibrillator + under medical supervision	2	3	8	7 DC Emery-Dreifuss muscular dystrophy (adult)
*FKRP*	LGMD2I/LGMD R9 Dystroglycan-related Muscular dystrophy-dystroglycanopathy (congenital with brain and eye anomalies), type A, 5 Muscular dystrophy-dystroglycanopathy (congenital with or without mental retardation), type B, 5	Curative treatment: frequent heart damage with proposed transplants, palliative treatment	2	1	6	
*FKTN*	LGMD2M/LGMD R13 Dystroglycan-related Muscular dystrophy-dystroglycanopathy (congenital with brain and eye anomalies), type A, 4 Muscular dystrophy-dystroglycanopathy (congenital without mental retardation), type B, 4	Curative treatment: frequent heart damage with proposed transplants, palliative treatment	2	1	6	
*FLAD1*	Lipid storage myopathy due to flavin adenine dinucleotide synthetase deficiency	Symptomatic treatment +/− riboflavine	2	1	6	
*FLNC*	Myopathy, distal, 4 Myopathy, myofibrillar, 5	Symptomatic treatment: physiotherapy, cardiological treatment	2	3	10	9 AD myopathy myofibrillar (adult)
*GAA*	Glycogen storage disease Type II (Pompe disease)-GSDII LGMD2V (Adult onset LGMD2 related to GAA deficiency)/Pompe disease	Specific treatment: enzyme replacement therapy (allergic reaction to enzyme replacement, less effective in early pediatric forms), IV/15 days	2	2	9	9 CB (adult)
*GBE1*	Glycogen storage disease type IV Polyglucosan body disease, adult form	Symptomatic treatment	2	1	4	
*GFPT1*	Congenital myasthenia 12, with tubular aggregates	Acetylcholinesterase inhibitors, 3,4-diaminopyridine, salbutamol, ephedrine	3	3	12	
*GMPPB*	Muscular dystrophy-dystroglycanopathy (congenital with brain and eye anomalies), type A, 14 Muscular dystrophy-dystroglycanopathy (congenital with mental retardation), type B, 14 LGMD2T	Acetylcholinesterase inhibitors, 3,4-diaminopyridine, salbutamol, ephedrine	2	3	11	
*HSPG2*	Dyssegmental dysplasia, Silverman-Handmaker type Schwartz-Jampel syndrome, type 1	Carbamazepine	2	3	10	
*ISCU*	Myopathy with lactic acidosis, hereditary	Symptomatic treatment: diagnosis is vital for handing emergency form to the patient (in case of rhabdomyolysis: hospitalized in ICU)	2	2	6	
*KCNA1*	Myokymia with or without episodic ataxia type 1	Acetazolamide to decrease severity attacks decrease +/− antiepileptic, physiotherapist	2	2	8	
*KCNJ2*	Andersen-Tawil syndrome	Acetazolamide, dichlorphenamide, antiarythmic therapeutics.Treatment and prevention of paralytic attacks, cardiac arythmias, malformation	3	3	11	
*KCNQ2*	Epileptic encephalopathy, early infantile, 7 Myokymia	Tegretol (depend of the type of mutation)	2	3	10	
*LAMB2*	Pierson syndrome	Palliative treatment, treatment of renal failure, Ephedrine for myasthenic syndrome	2	2	10	
*LMNA*	LGMD1B/Emery-Dreifuss muscular dystrophy Congenital muscular dystrophy due to LMNA defect	Symptomatic treatment: physiotherapist, orthopaedic surgery (tendon retraction), cardiological treatment, implantable Cardioverter-Defibrillator (ICD), Efficiency for preventing fatal ventricular tachycardia Heart transplantation	3	3	12	9-10 BN Dilated cardiomyopathy 7 DC Emery-Dreifuss muscular dystrophy
*MUSK*	Congenital myasthenic syndrome 9, associated with acetylcholine receptor deficiency Fetal akinesia deformation sequence	Salbutamol, +/− 3,4-Diaminopyridine/Ephedrine (partially effective)	3	3	12	
*PGM1*	PGM1-CDG (Congenital Disorder of Glycosylation)/Glycogen storage disease type XIV	Symptomatic treatment. Possible improvement with galactose intake reinforcing the importance of screening	2	1	4	
*PHKA1*	Glycogen storage disease type IXd (ex type VIII) or X-linked muscle phosphorylase kinase deficiency	Hygienodietic rules, physiotherapy	3	3	7	
*PHKB*	Glycogen storage disease type Ixb	Hygienodietic rules, physiotherapy	3	3	7	
*PLEC*	Congenital myasthenic syndrome with epidermolysis bullosa Epidermolysis bullosa simplex with muscular dystrophy LGMD2Q/LGMD R17 Plectin-related	Symptomatic treatment	2	3	11	
*PREPL*	Congenital myasthenic syndrome 22	Acetylcholinesterase inhibitor	2	3	11	
*PRKAG2*	Glycogen storage disease of heart, lethal congenital Cardiomyopathy, familial hypertrophic, with Wolff-Parkinson-white syndrome-CMH6	Prevention interest of arrhythmias and sudden death	3	2	8	10 NN cardiomyopathy familial hypertrophic
*PYGM*	Glycogen storage disease Type V (McArdle disease)	Measures to prevent episodes of rhabdomyolysis (avoid intense efforts) plus prevention in case of anesthesia and during pregnancies. Effectiveness on the phenomenon of the second wind of taking sugars by mouth before exercise	2	2	6	9 NC Glycogen storage disease V (adult)
*RAPSN*	Congenital myasthenic syndrome 11, associated with acetylcholine receptor deficiency Fetal akinesia deformation sequence	Acetylcholinesterase inhibitors, salbutamol	3	3	12	
*RYR1*	Susceptibility to malignant hyperthermia	Avoidance of triggering anesthetics	3	3	10	10 DB malignant hyperthermia susceptibility
*SCN4A*	Hyperkalemic periodic paralysis	Acetazolamide	3	3	9	
	Paramyotonia congenita	Mexiletine, carbamazepine	3	3	9	
	Potassium-aggravated myotonias (myotonia fluctuans)	Mexiletine, carbamazepine, acetazolamide	3	3	9	
	Potassium-aggravated myotonias (myotonia permanens, severe neonatal episodic laryngospasm)	Mexiletine, carbamazepine, acetazolamide	2	3	11	
	Congenital myasthenic syndrome type 16	Acetylcholinesterase inhibitors, acetazolamide	2	3	11	
*SLC22A5*	Primary systemic carnitine deficiency	L-carnitine per-os. Crucial importance of diagnosis as severe cardiomyopathy treatable by carnitine supplementation	2	2	7	
*SLC25A1*	Combined D-2- and L-2-hydroxyglutaric aciduria (Impaired neuromuscular transmission due to mitochondrial citrate carrier mutations)	Acetylcholinesterase inhibitors, 3,4-diaminopyridine	2	3	11	
*SLC25A32*	Riboflavin-responsive exercise intolerance (RREI)	Riboflavine plus symptomatic treatment	2	1	4	
*SLC5A7*	Congenital myasthenic syndrome 20, presynaptic Distal hereditary motor neuronopathy type VIIA	Acetylcholinesterase inhibitors, salbutamol	3	3	12	
*SNAP25*	Congenital myasthenic syndrome 18	3,4-Diaminopyrimidine	2	3	11	
*STIM1*	Tubular aggregate myopathy 1 Stormorken syndrome	Monitoring of Stormorken syndrome thrombopenia, haemorrhage, thrombosis	2	2	5	
*SYT2*	Congenital myasthenic syndrome 7, presynaptic (Lambert-Eaton myasthenic syndrome and nonprogressive motor neuropathy)	3,4-Diaminopyrimidine	2	3	11	
*TNNI2*	Distal arthrogryposis multiplex congenita type 2B	Symptomatic and palliative treatment (orthopedic surgery, physiotherapy, ergotherapy)	2	1	4	
*TNNT3*	Distal arthorgryposis type 2B	Symptomatic and palliative treatment (orthopedic surgery, physiotherapy, ergotherapy)	2	1	4	
*TRIP4*	Muscular dystrophy, congenital, davignon-chauveau type	Symptomatic treatment + orthopedic surgery	2	1	6	
*TRPV4*	Congenital distal spinal muscular atrophy, non progressive	Symptomatic and palliative treatment (orthopedic surgery, physiotherapy, ergotherapy)	2	1	4	
*TTN*	EOMFC-Salih myopathy Early-Onset Myopathy with Fatal Cardiomyopathy	Cardiac monitoring/pacemaker	2	3	8	
	Congenital myopathy with cores and cardiopathy	Cardiac monitoring/nocturnal ventilation/pacemaker	2	3	7	
	Congenital myopathy with central nuclei	Nocturnal ventilation/Cardiac monitoring	2	3	7	
	Emery-Dreifuss muscular dystrophy-like	Nocturnal ventilation/Cardiac monitoring	2	3	7	
	LGMD 2J/LGMDR10 Titin-related	Cardiac monitoring/pacemaker	2	3	8	
	HMERF	Cardiac monitoring and non-invasive ventilation	2	3	8	
	LGMD 2J/LGMDR10 Titin-related, with cardiomyopathy	Nocturnal ventilation/Cardiac monitoring	2	3	7	

## Data Availability

The data presented in this study are available on request from the corresponding author.

## Supplementary Materials

The supporting information can be downloaded at: www.mdpi.com/article/10.3390/ijms23158506/s1.
